# Learning rule sets from survival data

**DOI:** 10.1186/s12859-017-1693-x

**Published:** 2017-05-30

**Authors:** Łukasz Wróbel, Adam Gudyś, Marek Sikora

**Affiliations:** 1 Institute of Informatics, Silesian Univ. of Technology, Akademicka 16, Gliwice, 44-100 Poland; 2 Institute of Innovative Technologies, EMAG, Leopolda 31, Katowice, 40-189 Poland

**Keywords:** Survival analysis, Separate-and-conquer, Rule induction, Log-rank test, High throughput sequencing, Cancer

## Abstract

**Background:**

Survival analysis is an important element of reasoning from data. Applied in a number of fields, it has become particularly useful in medicine to estimate the survival rate of patients on the basis of their condition, examination results, and undergoing treatment. The recent developments in the next generation sequencing open new opportunities in survival study as they allow vast amount of genome-, transcriptome-, and proteome-related features to be investigated. These include single nucleotide and structural variants, expressions of genes and microRNAs, DNA methylation, and many others.

**Results:**

We present LR-Rules, a new algorithm for rule induction from survival data. It works according to the separate-and-conquer heuristics with a use of log-rank test for establishing rule body. Extensive experiments show LR-Rules to generate models of superior accuracy and comprehensibility. The detailed analysis of rules rendered by the presented algorithm on four medical datasets concerning leukemia as well as breast, lung, and thyroid cancers, reveals the ability to discover true relations between attributes and patients’ survival rate. Two of the case studies incorporate features obtained with a use of high throughput technologies showing the usability of the algorithm in the analysis of bioinformatics data.

**Conclusions:**

LR-Rules is a viable alternative to existing approaches to survival analysis, particularly when the interpretability of a resulting model is crucial. Presented algorithm may be especially useful when applied on the genomic and proteomic data as it may contribute to the better understanding of the background of diseases and support their treatments.

**Electronic supplementary material:**

The online version of this article (doi:10.1186/s12859-017-1693-x) contains supplementary material, which is available to authorized users.

## Background

Modeling the impact of covariates on survival time is an important task of survival analysis. The most popular approaches to this problem are parametric [1] and semi-parametric statistical techniques like Cox proportional hazards regression [2] and its extensions. However, restrictive assumptions made by these strategies and difficulty in representing nonlinear interactions between covariates are one of the motivations for developing new methods based on machine learning techniques. The application of machine learning to survival analysis usually allows overcoming the limitations of statistical methods. In this paper we investigate a nonparametric rule-based approach to modeling survival data.

Rule induction is one of the oldest and most frequently used methods of machine learning. Although numerous successful applications in a wide range of predictive and descriptive data mining tasks, there is still a little research on rule learning in survival analysis. Naturally, in the case of absence of censored observations the standard rule-based regression [3–5] techniques can be applied. However, as the overwhelming majority of survival datasets contains censored instances, the methods able to handle censored data are of great value. In this paper we investigate rule induction algorithm in combination with the log-rank statistical test [6]. This nonparametric test is used to compare the survival distributions of two samples and is appropriate for censored data analysis. In our study the test is used to establish the key factors affecting overall survival time of observations covered by the rules being induced. As the basis of rule induction method we selected a separate-and-conquer (known also as covering) strategy [7, 8] which is one of the most common heuristics for induction of classification rules.

### Related work

Methods of survival analysis are mainly used in medical studies. Although rule-based algorithms are often applied in medical research, there is a relatively small number of papers concerning the application of rule induction to survival analysis.

Pattaraintakorn and Cercone [9] describe the rough set-based intelligent system for survival analysis. The model construction relies on a so-called minimal decision rule induction algorithm for identification of the main factors affecting survival time of patients. The survival time is considered as a discrete variable with predefined values (e.g. survival time between 56 and 73 months) dividing an entire dataset into separate decision classes.

The rough set-based approach to survival analysis is also the subject of the Bazan et al.’s work [10]. For each observation in the analyzed dataset, a prognostic index (PI) based on the Cox’s proportional hazard model is calculated. A range of PI values is divided into three intervals, thereby creating separate groups differing in the survival rate, and the rules are induced for resulting classes.

Sikora et al. [11] applied rule induction algorithm to the analysis of patients after bone marrow transplantation. The set of patients is divided into three groups: the patients for whom at least 5 years have passed since the transplantation (the class *alive*), the patients who died within 5 years after transplantation (the class *dead*), and the patients who are still alive but their survival time is less than 5 years (the class *alive-5*). Rules are generated for dataset containing *alive* and *dead* classes, whereas the *alive-5* is used for the post-processing of obtained rules.

Kronek and Reddy [12] proposed the extension of Logical Analysis of Data (LAD) [13, 14] for survival analysis. The LAD algorithm is a combinatorial approach to rule induction. It was originally developed for the analysis of data containing binary attributes, therefore the data pre-processing by discretization and binarization methods is usually required.

Liu et al. [15] adapted patient rule induction method to the analysis of survival data. The method uses so-called bump hunting which creates rules by searching regions in covariates space with a high average value of the target variable. To deal with censoring, the authors use deviance residuals as the outcome variable. The idea of residual-based approach to censored outcome is derived from survival trees [16, 17].

Wróbel [18] proposed to use a survival tree for induction of an ordered set of rules (decision list) from survival data. The core idea is to learn a survival tree, extract the best rule from it, and remove observations which are covered by the rule. The procedure is recursively repeated for remaining observations. This idea follows the approach used by the PART [19] and M5Rules [3] algorithms for learning classification and regression rules, respectively.

Wróbel and Sikora [20] investigated a separate-and-conquer method of rule induction in combination with a weighting scheme for handling censored observations. Each observation is assigned an appropriate weight to a positive or negative class. The positive class represents observations with high risk of event occurrence, whereas negative class includes potentially event-free ones. If observation have experienced an event, then it belongs to the positive class with weight equal to 1. Censored instances are assigned to both classes, but with different weights. The observations censored earlier receive higher weight for the positive class than the observations censored later. In the experimental study the authors pay special attention to rule quality measures [21–23] which are one of the key elements of rule induction algorithms.

It should be noted that the aforementioned studies primarily concern the application of rule-based survival analysis to usually one, particular dataset. Pattaraintakorn and Cercone [9] mainly focused on geriatric data of Canadian patients, Bazan et al. [10] analyzed data of patients with various kinds of the head and neck cancer cases, Sikora et al. [11] studied the effects of bone marrow transplantation, Liu et al. [15] performed an analysis of kidney cancer tissue microarray data. Kronek and Reddy [12] proposed a more general approach, however they verified the algorithm for only two real-life datasets. The exceptions are our previous work [18, 20] where survival tree-based and weighted separate-and-conquer algorithm for rule induction were tested on over a dozen various survival datasets.

There are also machine learning methods dedicated to censored data analysis and not associated with the rule induction. These are trees [16, 24–26], neural networks [27–29], bayesian networks [30, 31], support vector machines (SVM) [32], and ensemble approaches [33–35]. Among all aforementioned methods, the most widely used are tree-based techniques called survival trees.

Survival trees are an adaptation of classification and regression trees [36] to the problem of survival. In comparison to rule-based techniques, tree-based methods received much more attention in survival analysis [26]. On the other hand, a tree can be easily represented in the form of a set of rules where each path from the root to the leaf of the tree corresponds to one rule, thus it can be considered as a special case of the rule-based model. The key idea of the application of tree-based techniques to survival data lies in the splitting criterion [37]. The most popular approaches are residual-based ones [16, 17] as well as methods employing log-rank statistics [25, 38] for the maximization of the difference between survival distributions of child nodes. While searching for optimal splitting point with the use of the log-rank criterion, resampling methods are used too [39]. The extension of the decision trees idea are decision tree ensembles which includes, for example, bagging [40] and random forests [41]. The survival trees are also commonly employed in ensemble methods like bagging [35, 42], boosting [33] and random forests [34, 43, 44]. An extensive review and discussion on the induction of survival trees and survival tree ensembles can be found in [45]. In this work the merits and limitations of these methods are discussed, along with the available computer software.

One of important aspects of using the survival analysis in medical sciences and bioinformatics is the necessity to have easily interpretable results. This ability is a crucial feature of survival trees and survival rules. Both approaches divide the observations into subgroups with different survivability characteristics. Importantly enough, they allow not only the attributes that have significant impact on the survival time to be identified, but also non-linear dependencies and interactions between the variables to be modelled.

While survival trees can be straightforwardly translated to survival rules, the algorithms used for induction of the latter directly from data have numerous advantages. Firstly, divide-and-conquer (DnC) tree generation strategy forbids examples to be covered by multiple rules. Separate-and-conquer (SnC) heuristics for rules induction lacks this limitation often leading to discovering stronger or completely new dependencies in the data. Secondly, generation of rules from the tree by following the path from the root to leafs results in condition redundancy. This is not the case in SnC, as each rule is induced separately. The last feature is also useful when it is necessary to modify the generated rules so that they could better correspond to the domain knowledge. The SnC-generated rules can be a preliminary set of hypotheses which is then verified by an analyst (domain expert). By adding or deleting elementary conditions from the rules, or modifying their ranges, the analyst can carry out different variants of the analysis. Consequently, adding new rules to the set does not interact with existing ones. The tree, in contrast, should be treated as a whole. Therefore, a change of a condition in a tree node involves the need to modify or re-calculate the conditions in all its child nodes.

### Objectives and outline

The main goal of this paper is to present the separate-and-conquer rule learning algorithm designed for survival data analysis and to verify its effectiveness on the variety of survival problems. In contrast to most of the aforementioned related work, we propose a more general solution rather than the case-study approach. Moreover, as opposed to [9, 10, 12], the presented strategy does not require data pre-processing with the use of discretization methods. It is particularly important for the quality of survival analysis because discretization may cause the loss of information, and the final performance of the model may strongly depend on a selected discretization technique.

The key feature of our algorithm is the use of the separate-and-conquer strategy and log-rank statistical test for supervising the rule induction process. The log-rank test is aimed at detecting the most powerful and important factors affecting the expected survival time. Therefore, the resulting rule-based data models should be concise, easy to interpret by domain experts, and accurate in the survival time prediction. The use of the log rank-test requires neither the weight assignment to examples nor defining decision classes (e.g. event, non-event). All of these features distinguish the presented algorithm from the other approaches.

The efficiency of our rule-based framework for survival analysis was verified on a collection of 18 survival datasets describing a wide variety of real-life medical and biological problems. We compared our solution with the state-of-art survival trees algorithms.

In addition, we present the detailed analysis of rules sets for German Breast Cancer Study Group 2 [46], Bone Marrow Transplantation [47], Lung Adenocarcinoma [48], and Papillary Thyroid Carcinoma [49] datasets. The results show that the rule-based models generated by our algorithm are useful and can provide interesting information about the data, particularly when faced with the recent development of bioinformatics technologies.

The algorithm is available at http://www.adaa.polsl.pl/software.html.

## Methods

Let *D*(*A,T*,*δ*) be the survival dataset of |*D*| observations (examples, instances). Each example is characterized by a set of covariates (attributes) *A*={*A*_1_,*A*_2_,…,*A*_|*A*|_}, an observation time *T*, and a censoring status *δ*. Therefore, *i*-th example can be represented as a vector *o*_*i*_=(*a*_*i*1_,…,*a*_*i*|*A*|_,*T*_*i*_,*δ*_*i*_). In the study we consider right-censored data model which is the most common in the survival analysis. Consequently, *T*_*i*_ denotes either the time of the observation for event-free examples (*δ*_*i*_=0) or the time before the occurrence of an event (*δ*_*i*_=1).

The LR-Rules algorithm returns a set of survival rules. A survival rule *r* has the form:

$$\mathbf{IF}\ c_{1}\ \pmb{\wedge}\ c_{2}\ \pmb{\wedge}\ \ldots\ \pmb{\wedge}\ c_{n}\ \mathbf{THEN}\ \hat{S}(T|c_{j}) $$

The premise of the rule is a conjunction of conditions. If attribute *A*_*j*_ is of nominal type, condition *c*_*j*_ has the form *A*_*j*_=*a*_*j*_; if *A*_*j*_ is numerical, *A*_*j*_<*a*_*j*_ or *A*_*j*_≥*a*_*j*_ conditions are possible (with *a*_*j*_ being an element of the *A*_*j*_ domain). An observation is covered by the rule when it satisfies its premise. The conclusion of *r* is an estimate $\hat {S}(T|c_{j})$ of the survival function. Particularly, it is a Kaplan-Meier (KM) estimator [50] calculated on the basis of the instances covered by the rule, that is, satisfying all conditions *c*_*j*_ (*j*=1,…,*n*).

The induction of survival rules in LR-Rules follows the separate-and-conquer heuristics. The algorithm adds rules iteratively to the initially empty set. Every learned rule has to cover at least *mincov* previously uncovered examples from the input dataset. The iteration continues until entire dataset becomes covered by the rule set. The pseudocode of the separate-and-conquer approach is presented in Algorithm 1.

The aim of the induction algorithm is to obtain rules of maximum quality. An extensive research on classification rules [21–23] showed that proper selection of a quality measure is of crucial importance for comprehensibility and performance of output model. In the survival analysis it is desirable for a rule to cover examples which survival distributions differ significantly from that of other instances. In presented algorithm, KM survival estimates of the examples covered and uncovered by the rule are derived from the data. A log-rank test statistics for those estimates is then used as a quality measure. The log-rank statistics is calculated as *x* ^2^/*y* where:

$$\begin{array}{*{20}l} x = & \sum\limits_{t \in T_{c} \cup T_{u}} \left(d^{t}_{u} - \frac{r^{t}_{u}}{r^{t}_{c} + r^{t}_{u}} \cdot \left(d^{t}_{c} + d^{t}_{u}\right)\right)\\ y = & \sum\limits_{t \in T_{c} \cup T_{u}} \frac{r^{t}_{c} \cdot r^{t}_{u} \cdot \left(d^{t}_{c} + d^{t}_{u}\right) \cdot \left(r^{t}_{c} + r^{t}_{u} - d^{t}_{c} - d^{t}_{u}\right)}{\left(r^{t}_{c} + r^{t}_{u}\right)^{2} \cdot \left(r^{t}_{c} + r^{t}_{u} - 1 \right)} \end{array} $$

*T*_*c*_ and *T*_*u*_ are sets of event times of observations covered and not covered by the rule, $d_{c}^{t}\left (d_{u}^{t}\right)$ is the number of covered (uncovered) observations which experienced an event at time *t*, and $r_{c}^{t}\left (r_{u}^{t}\right)$ is the number of covered (uncovered) instances at risk, that is, which are still observable at time *t*.









The induction of a rule is performed in two stages: growing and pruning. The former consists in greedy addition of elementary conditions to the initially empty rule premise (Algorithm 2). At each step, the algorithm searches exhaustively for the condition whose addition renders rule of the highest quality. If several conditions lead to the same value of the log-rank statistics, the one covering more examples is selected. The set of all the possible conditions which might be added to the rule is created on the basis of examples currently covered by the rule (Algorithm 3). In the case of nominal attributes, conditions in the form *A*_*j*_=*a*_*j*_ for all values *a*_*j*_ from the attribute domain are considered. For continuous attributes, *A*_*j*_ values that appear in the observations covered by the rule are sorted. Then, the possible split points *a*_*j*_ are determined as arithmetic means of adjacent elements and conditions *A*_*j*_<*a*_*j*_ and *A*_*j*_≥*a*_*j*_ are evaluated. Te prevent from generation of too specific rules, conditions whose addition would cause the rule to cover less than *mincov* previously uncovered examples are discarded. The growing stops when no conditions satisfying aforementioned criterion remain.

Growing stage is followed by pruning (Algorithm 4). The procedure iteratively removes conditions from the premise, each time making an elimination leading to the largest improvement in the quality. The procedure stops when no conditions can be deleted without decreasing the log-rank statistics or when rule contains only one condition.

Finally, for comprehensibility, the output rules are post processed by merging conditions based on the same numerical attributes. For example, the conjunction: *A*_*i*_≥*x* ∧ *A*_*i*_<*y* is transformed into a single condition *A*_*i*_∈〈*x,y*).

**Fig. 1:**
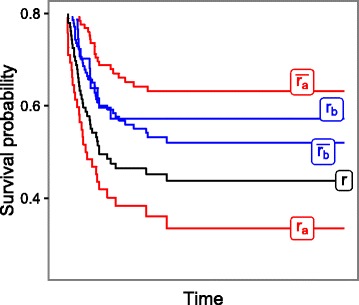
Growing a survival rule supervised by the log-rank criterion. Among two possible refinements *r*_*a*_ and *r*_*b*_ of the rule *r*, the *r*_*a*_ is selected as it maximizes the difference between survival curves of the observations covered and not covered by the rule (lines labelled with *r*_*a*_ and $\overline {r_{a}}$, respectively)

Figure 1 illustrates the idea of rule growing supervised by the log-rank criterion. Let *r* be the input rule with two possible refinements *r*_*a*_ and *r*_*b*_. The figure shows the KM curves of all these rules. Additionally, the graph presents the survival curves of the observations not covered by the rules *r*_*a*_ and *r*_*b*_, labelled with $\overline {r_{a}}$ and $\overline {r_{b}}$, respectively. The log-rank statistics calculated for the rule *r*_*a*_ (*r*_*b*_) reflects difference between survival curves labelled with *r*_*a*_ (*r*_*b*_) and $\overline {r_{a}}$ ($\overline {r_{b}}$). The difference between *r*_*a*_ and $\overline {r_{a}}$ is greater than for the pair *r*_*b*_–$\overline {r_{b}}$. Therefore, the refinement *r*_*a*_ of the rule *r* better discriminates observations according to the survival rate, thus it is selected as the current best form of the rule which is expanded with new conditions in the subsequent iterations.

In order to deal with missing attribute values, LR-Rules employs an ignored value strategy in which rules are built based only on known values of observations. It is performed straightforwardly by skipping missing values during search of possible conditions. The observation having a missing value of an attribute tested by the rule is considered to be uncovered by this rule. In contrast to imputation methods [51], this strategy does not require any additional computations and, as was shown in [52], it performs similarly to more advanced and computationally expensive approaches to handling missing values.

A valuable property of LR-Rules is also the ability to handle datasets with weighted observations. In this case, the value of log-rank test is calculated on the basis of weights and *mincov* parameter indicate the sum of observations weights to be covered by a newly generated rule.

**Fig. 2:**
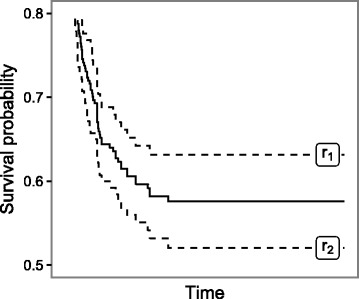
Averaging survival curves. When the observation is covered by multiple rules (*r*_1_ and *r*_2_ in this case), its survival function (solid line) is obtained as an average of the rule functions (*dashed lines*)

The learned rule set can be applied for an estimation of the survival function of new observations based on the values taken by their covariates. The estimation is performed by rules covering given observation. If observation is not covered by any of the rules then it has assigned the default survival estimate computed on the entire training set. Otherwise, final survival estimate is calculated as an average of survival estimates of all rules covering the observation (see Fig. 2 for an example).

## Results and discussion

### Experimental setting

The LR-Rules algorithm was investigated on 18 sets listed in Table 1 using 10-fold stratified cross-validation repeated ten times for each set. The stratification of survival data was performed according to the censoring status, that is, the proportion of events to censored observations in each fold was the same as in the entire training set. Additionally, the detailed analysis of survival rules was performed on four selected sets. These were GBSG2 (German Breast Cancer Study Group 2) [53], BMT-Ch (Bone Marrow Transplantation – Children) [20, 47], LAC (Lung Adenocarcinoma) [48], and PTC (Papillary Thyroid Carcinoma) [49].

**Table 1:** The characteristics of 18 sets used in the experimental studies: the number of observations (#obs), the number of conditional attributes (#att), the percentage of missing values (%mv), the percentage of censored observations (%cs), and the research subject

Set	#obs	#att	%mv	%cs	Subject of research
actg320 [63]	1151	11	0	92	HIV-positive patients
BMT-Ch [47]	187	37	1	55	Bone marrow transplant
cancer [64]	228	7	4	28	Advanced lung cancer
follic [65]	541	4	0	36	Follicular lymphoma
GBSG2 [53]	686	8	0	56	Breast cancer
hd [65]	865	6	0	51	Hodgkin’s disease
LAC [48]	86	113	0	72	Lung adenocarcinoma
lung [66]	1032	7	3	26	Early lung cancer
Melanoma [67]	205	7	0	65	Malignant melanoma
mgus [68]	241	9	20	24	Monoclonal gammopathy
PTC [49]	421	24	41	93	Papillary thyroid carcinoma
pbc [69]	418	17	15	61	Primary biliary cirrhosis
std [70]	877	21	0	60	Sexually-trans. diseases
uis [63]	575	13	0	19	Drug addiction treatment
wcgs [71]	3154	10	<1	92	Coronary artery disease
whas1 [63]	481	7	0	48	Myocardial infarction ed1
whas500 [63]	500	13	0	57	Myocardial infarction ed2
zinc [72]	431	55	57	81	Esophageal cancer

GBSG2 is a well-known dataset which describes patients with primary node positive breast cancer. It was used, inter alia, in [12, 31, 39] to test different modeling techniques. Each observation is described by the following attributes: hormonal therapy (*horTh*), age, menopausal status (*menostat*), tumour size (*tsize*), tumour grade (*tgrade*), number of positive nodes (*pnodes*), progesterone receptor (*progrec*), estrogen receptor (*estrec*). An event in survival analysis is cancer recurrence.

BMT-Ch describes 187 patients (75 females and 112 males) at the age of 0.6 to 20.2 years (median 9.6) admitted to the Department of Pediatric Bone Marrow Transplantation, Oncology and Hematology, Wrocław Medical University, Poland. Disease spectrum included 155 malignant disorders (i.a. 67 patients with acute lymphoblastic leukemia, 33 with acute myelogenous leukemia, 25 with chronic myelogenous leukemia, 18 with myelodysplastic syndrome) and 32 nonmalignant cases (i.a. 13 patients with severe aplastic anemia, 5 with Fanconi anemia, 4 with X-linked adrenoleukodystrophy). The procedure of unmanipulated allogeneic unrelated donor hematopoietic stem cell transplantation was performed in each case, according to the European protocols or the guidelines of the European Blood and Marrow Transplant Inborn Errors Working Party with worldwide accepted modifications based on disease and/or patient’s condition status prior transplantation. Each patient was characterized by a set of 42 conditional attributes. Table 2 presents interpretations of selected ones. Patient’s death is considered as an event.

**Table 2:** Selected conditional attributes of BMT-Ch (Bone Marrow Transplantation) dataset

Name	Description
*RecipientRh*	Presence of the Rh factor on recipient’s red blood cells
*RecipientAge*	Age of the recipient of hematopoietic stem cells at the time of transplantation
*RecipientBodyMass*	Body mass of the recipient of hematopoietic stem cells at the time of transplantation
*CMV_status*	Serological compatibility of the donor and the recipient of hematopoietic stem cells according to cytomegalovirus infection prior to transplantation
*RecipientABO*	ABO blood group of the recipient of hematopoietic stem cells
*DonorABO*	ABO blood group of the donor of hematopoietic stem cells
*ABOmatch*	Compatibility of the donor and the recipient of hematopoietic stem cells according to ABO blood group
*DonorAge*	Age of the donor at the time of hematopoietic stem cells apheresis
*HLAmatchCompatibility*	Compatibility of antigens of the main histocompatibility complex of the donor and the recipient of hematopoietic stem cells (10/10, 9/10, 8/10, 7/10 allele/antigens) according to ALL international BFM SCT 2008 criteria
*Relapse*	Reoccurrence of the disease
*GvHD_III_IV*	Development of acute graft versus host disease stage III or IV
*extcGvHD*	Extensive chronic graft versus host disease
*CD34* (10^6^/kg)	CD34+ cell dose per kg of recipient body weight
*CD3* (10^8^/kg)	CD3+ cell dose per kg of recipient body weight
*CD3/CD34*	CD3+ cell to CD34+ cell ratio

LAC dataset concerns gene expression profiles of 86 lung cancer patients [48]. Expressions were measured with Affymetrix hu6800 microarrays (7 129 probe sets) and normalized from raw.CEL files by RMAExpress. In the experiments we considered 100 genes with greatest effect on survival rate according to Beer et al. [48]. Due to name discrepancies, three genes were excluded from the investigation as they did not map to any probe. On the other hand, some genes had multiple probes assigned. As a result, LAC dataset contains 113 conditional attributes with patient’s death being considered as an event.

PTC gathers information about 492 papillary thyroid cancer patients. They are characterized by clinical as well as genome-related features like single nucleotide polymorphisms (SNP), copy number alterations (CNA), gene expressions determined with RNA-seq, DNA methylation, protein expressions obtained by reverse phase protein arrays (RPPA), etc. Data table available at [54] was processed by filtering out patients with missing information about survival status or survival time. As we wanted to focus this study on the genetic background of thyroid cancer, corresponding features were selected for further analysis (Table 3). We assumed recurrence of a cancer to be an event in the survival analysis.

**Table 3:** Selected conditional attributes of PTC (Papillary Thyroid Carcinoma) dataset

Name	Description
*BRAFV600ERAFClass*	Flag indicating if tumor is driven by BRAF or RAS genes
*BRAFV600E_RAS_score*	Continuous score from (−1,1) interval describing to what extent a tumor expression profile resembles BRAF- or RAS-mutant profiles
*mRNA_cluster_number*	Number of mRNA expression cluster (1–5)
*miRNA_cluster_number*	Number of microRNA expression cluster (1–6)
*RPPA_cluster_number*	Number of protein expression cluster (1–4)
*meth_cluster*	DNA methylation pattern (one of four)
*Arm_SCNA_cluster*	Chromosomal arm-level copy number alterations pattern (one of four)
*nmut_APOBEC*	Mutation density (mutations/Mb) associated with APOBEC cytidine deaminases
*nmut_CpGT*	Mutation density (mutations/Mb) of CpG islands
*person_gender*	Gender
*race_category*	Race (Black/White/Asian/American Indian)
*ethnicity_category*	Ethnicity (Hispanic/Non-Hispanic)

The results of the LR-Rules algorithm were compared with results achieved by the KM estimator, our earlier CW-Rules algorithm [20], and two implementations of survival trees (CTREE, RPART). The CTREE algorithm [39] builds model from survival data using a splitting criterion based on the log-rank statistic. The RPART algorithm [55] fits time variable into exponential model, and then it applies Poisson regression to such modified data. It leads to method equivalent to the deviance residual-based approach of LeBlanc and Crowley [16].

The performance of rule sets was evaluated with a use of the integrated Brier score (IBS) [56, 57]. The Brier score at time *T* ^⋆^ for i-th observation is given by:

$$BS_{i}(T^{\star}) = \left\{\begin{array}{ll} \frac{1}{\hat{G}(T_{i})}\cdot[0 - \hat{S}(T^{\star})]^{2} & \text{if \(T_{i} \le T^{\star}\), \(\delta_{i} = 1\)} \\ \frac{1}{\hat{G}(T^{\star})}\cdot[1 - \hat{S}(T^{\star})]^{2} & \text{if \(T_{i} > T^{\star}\)} \\ 0 & \text{in other cases} \end{array}\right. $$

The Brier score *BS*_*i*_(*T* ^⋆^) represents the squared difference between true event status at time *T* ^⋆^ and predicted event status $\hat {S}(T^{\star })$ at that time. The true event status for i-th observation is equal to 0 if an event occurred for this observation before or at the time *T* ^⋆^, and it is equal to 1 if survival time *T*_*i*_ of the observation is greater than *T* ^⋆^. The censoring is taken into account by weighting the squared differences by the inverse of the estimate $\hat {G}$ of the censoring survival function. The $\hat {G}$ estimate is calculated as the KM estimator based on training observations with censoring status set to (1−*δ*). If observation was censored before time *T* ^⋆^ then its weight is equal to 0. However, such observations have indirect contribution to final score because they are considered in calculation of $\hat {G}$ estimate.

The IBS summarizes the prediction error over all *n* observations and over all times in a test set:

$${IBS} = \frac{1}{\max{T_{i}}} \int_{0}^{\max{T_{i}}}BS(T^{\star})dT^{\star} $$

where

$$BS(T^{\star}) = \frac{1}{n} \sum_{i=1}^{n}BS_{i}(T^{\star}) $$

Lower IBS values correspond to better prediction accuracy.

In the experimental study, the algorithms were compared on multiple datasets with the use of statistical tests recommend by Demšar [58]. For comparison of two algorithms on multiple datasets we used the Wilcoxon signed rank test, while the comparisons of all algorithms with each other were preformed with a use of the Friedman test followed by the post-hoc Nemenyi test.

### Experimental evaluation

The first experimental step was to investigate the influence of *mincov* parameter on the results of the LR-Rules algorithm. This parameter specifies the minimum number of uncovered observations that must be covered by a newly generated rule during the growing phase. The minimum value of this parameter is 1, which corresponds to the case when each induced rule must cover at least one yet uncovered example. The greater the value of *mincov*, the higher is the coverage of resulting rules. This decreases the cardinality of the final rule set.

In the study, *mincov* values ranging from 1 to 7 were examined. The upper bound of seven was selected as this is a default value of the *minbucket* parameter, which defines the minimum number of observations in the leaves of CTREE and RPART trees. Detailed results, i.e., Brier scores and numbers of rules for different *mincov* values are presented in Additional file 1: Tables S1 and S2.

The analysis of *mincov* effect on IBS with a use of Friedman test, revealed that at least one of the investigated parameter values generated models of significantly different accuracy than the others (*p*-value of 0.0478). However, the results of the Nemenyi’s post-hoc test (summarized in Additional file 1: Figure S1 as a critical difference diagram) showed no statistical significance at 0.05 level.

**Fig. 3:**
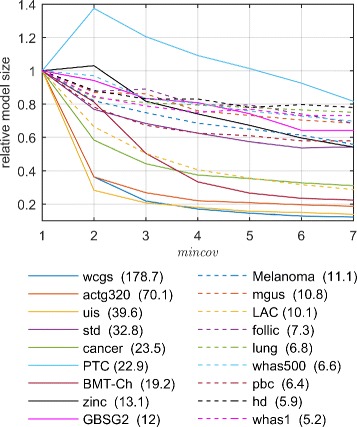
Influence of the *mincov* parameter on the LR-Rules model size. The model size for each dataset is defined as the number of rules normalized by the number of rules for *mincov*=1 (given in the legend)

The different situation was in the case of the size of resulting rule sets. As presented in Fig. 3, increasing *mincov* parameter caused noticeable reduction in the number of rules. Importantly enough, the greater the initial model, the larger decrease was observed. The comparison of parameter values with a use of the Friedman test rejected the null hypothesis about all parameter values generating same number of rules with *p*-value close to zero. A summary of the Nemenyi post-hoc test (Additional file 1: Figure S2) revealed the lack of significance only within groups of three neighbouring *mincov* values. The strong dependency between *mincov* and the model size was also confirmed statistically: the Pearson’s correlation between the parameter value and the rank was close to −1.0.

Setting *mincov* parameter to 7 resulted in the most compact models: for the majority of survival datasets containing hundreds of observations, the algorithm generated less than eight rules. For this reason, and due to lack of significant effect of the parameter on the accuracy, 7 was set as the default *mincov* value in LR-Rules and was used in further experiments, unless specified otherwise.

**Fig. 4:**
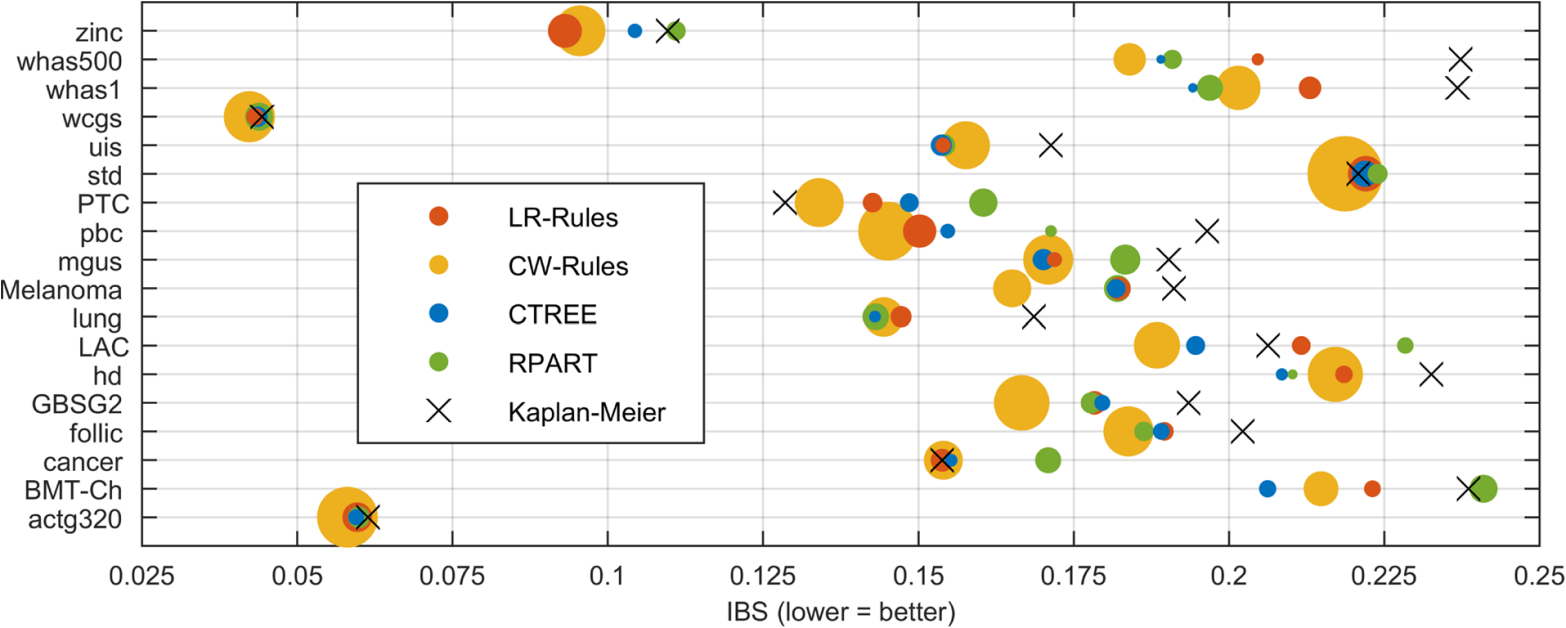
Comparison of the algorithms on the investigated datasets. Horizontal axis corresponds to the prediction accuracy (IBS), bubble diameters are proportional to the logarithm of the number of rules

The next part of the study was to compare LR-Rules to CW-Rules, CTREE, RPART, and the KM estimator in terms of the accuracy and the model size. The results for particular datasets are presented in Fig. 4 as bubbles with horizontal coordinates corresponding to IBS (lower = better) and diameter proportional to the logarithm of the number of rules. The results in the numerical form can be found in Additional file 1: Tables S3 and S4.

**Fig. 5:**
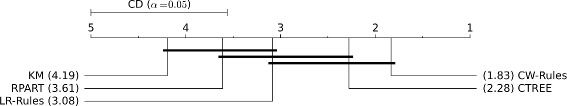
Statistical analysis of the prediction accuracy. Critical difference diagram comparing LR-Rules, CW-Rules, CTREE, RPART algorithms, and the KM estimator in terms of the integrated Brier score (IBS) at the significance level 0.05 over 18 datasets. Average ranks are shown in parentheses (lower = better). The groups of algorithms which are not significantly different are connected with *bold lines*

The Friedman test showed statistically significant differences between the LR-Rules, CW-Rules, CTREE, RPART and KM algorithms in terms of the IBS criterion (*p*-value <10^−4^). The visualization of Nemenyi’s post-hoc test at the 0.05 significance level is presented in Fig. 5. LR-Rules was in the group of three best algorithms together with the CW-Rules and CTREE. The worst results were obtained by the KM estimator. Interestingly, the Nemenyi’s test indicated no difference between KM, RPART and LR-Rules. Nevertheless, as this test is often too conservative to show the difference [59], an additional comparison between LR-Rules and the competitors was carried out using the Wilcoxon test with the Finner correction [60]. The test showed our algorithm to be superior to the KM estimator in terms of IBS (*p*_corrected_=0.0062). In contrast, the comparison with CTREE and RPART revealed lack of significance (both uncorrected and corrected *p*-values were noticeably greater than 0.05). CW-Rules achieved lower prediction error on the investigated data than LR-Rules (corrected *p*-value equaled to 0.0051).

As Additional file 1: Table S4 shows, superior accuracy of CW-Rules was obtained at the cost of the model size: for all analyzed datasets it generated several times greater rule sets than other methods. This was confirmed by the statistical analysis. While LR-Rules, CTREE, and RPART generated models of similar complexity (lack of significance at 0.05 level), CW-Rules induced significantly more rules (Additional file 1: Figure S3).

Table 4 provides detailed characteristics of the models generated by LR-Rules. The output rules usually contained from 1 to 7 elementary conditions, but majority of them had at most 3 conditions. Each of rules covered on average 36% of the observations from the training set. Importantly, the greater the number of rules in a set, the lower the coverage: the Pearson correlation coefficient between those variables equaled to −0.9135. The significance of rules was assessed statistically by performing log-rank test between Kaplan-Meier estimators of observations covered and uncovered by investigated rule. To control false discovery rate, the Benjamini-Hochberg correction was applied [61]. As it is shown in Table 4, the percentage of statistically significant rules at 0.05 level was close to 100%.

**Table 4:** The characteristics of rule sets generated by LR-Rules: the value of the integrated Brier score (IBS), the number of generated rules (#rules), the average rule length, the average rule coverage (%cov), a percentage of significant rules (*p*-value of log-rank test with FDR adjustment below 0.05; %sign)

Dataset	IBS	#rules	Length	%cov	%sign
actg320	0.0597	13.1	3.7	28	99.18
BMT-Ch	0.2231	4.3	3.3	48	98.40
cancer	0.1538	7.3	3.4	40	97.35
follic	0.1896	5.0	1.8	38	99.32
GBSG2	0.1783	7.7	2.7	36	99.75
hd	0.2185	4.6	1.3	44	99.43
LAC	0.2116	2.8	6.8	41	100.00
lung	0.1472	5.1	1.0	45	100.00
Melanoma	0.1823	6.2	2.6	33	99.69
mgus	0.1719	7.4	3.2	34	99.70
PTC	0.1426	18.7	3.0	22	79.69
pbc	0.1502	3.7	1.1	45	100.00
std	0.2220	17.8	6.5	25	98.74
uis	0.1539	5.5	1.2	41	100.00
wcgs	0.0432	21.8	5.2	19	98.95
whas1	0.2130	3.8	1.3	43	100.00
whas500	0.2046	4.6	1.2	40	100.00
zinc	0.0931	7.1	2.5	31	98.42

### Case studies

In order to demonstrate the rules induced by the presented algorithm, the detailed analysis of GBSG2, BMT-Ch, LAC, and PTC was performed. To obtain the most comprehensible models for the investigated datasets, *mincov* parameter was set to 3, 5, 7, and 12, respectively.

The rule set induced by the algorithm for the whole GBSG2 dataset consisted of 10 rules. Four of them are presented below:

- **R1**: *progrec* ≥ 108.0
- **R2**: *pnodes* < 5.5 ∧*progrec* ≥ 16.5 ∧*age* ≥ 39.5
- **R3**: *pnodes* ≥ 4.5 ∧*progrec* < 23 ∧*age* ∈ [41.5, 59.5) ∧*estrec* ∈ [0.5, 37.0)
- **R4**: *pnodes* ≥ 4.5 ∧*progrec* < 28.5

**Fig. 6:**
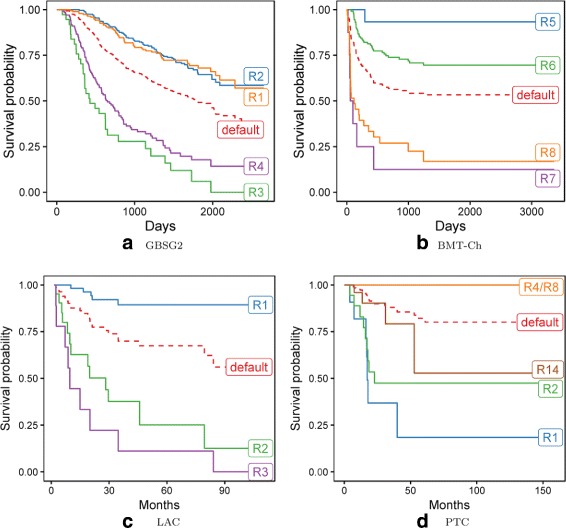
Analysis of the rules induced for GBSG2 (**a**), BMT-Ch (**b**), LAC (**c**), and PTC (**d**) datasets. The charts demonstrate the Kaplan-Meier survival curves for the observations covered by selected rules. The dashed (*default*) curves represent the KM estimates of the entire datasets

The KM survival curves for observations covered by the R1-R4 rules are presented in Fig. 6 a. The graph additionally includes a *default* curve representing the KM estimate for the entire GBSG2 dataset. The significant difference can be observed between the survival estimates determined by R1-R2 rules, which are above the default estimate, and R3-R4, which are placed below. Neither of the 10 induced rules had *horTh* attribute, indicating that the patient was a subject to the hormonal therapy. This result is consistent with the conclusions of the work [46], stating that: *No significant difference in recurrence-free survival was observed with respect to hormonal therapy*.

The rule set induced by the algorithm for the entire BMT-Ch data consisted of 7 rules. The motivation of this study was to identify the most important factors influencing the success or failure of the transplantation procedure. In particular, verification of the research hypothesis that increased dosage of CD34+ cells/kg extends overall survival time without simultaneous occurrence of undesirable events affecting patients’ quality of life [11, 47]. Four of the induced rules are presented below:

- **R5**: *DonorAge* ∈ [31, 41.7) ∧*CD34* ≥10·10^6^
    ∧*CD3/CD34* ≥ 3.4 ∧*RiskGroup* = Low
    ∧*RecipientBodyMass* < 69.5
- **R6**: *extcGvHD* = No
- **R7**: *DonorABO* = 0+ ∧*Relapse* = No
    ∧*CD34* <11.84·10^6^∧*CD3/CD34* ≥ 6.83
- **R8**: *DonorAge* ≥ 20.4 ∧*CD34* ≤ 10
    ∧*RecipientAge* ∈ [14.05, 19.5)

Figure 6 b presents the KM survival curves for observations covered by the R5-R8 rules, as well as the *default* estimate for the entire dataset. As in the previous case, the R5-R6 curves are above the default estimate, while R7-R8 are below.

The *CD34* attribute occurred often in the induced rules. It can be seen that lower doses of the *CD34* cells affected the shorter survival time, while higher doses increased this time. In the paper [47] the impact of *CD34* doses on the overall survival time was analyzed by dividing the value of *CD34* into two intervals: ≤10 and >10. The rules induced by the proposed algorithm are consistent with [47] and they additionally clarify the conditions under which the doses of *CD34* are even more important for the survival time. It should also be noted that the rule R6 states that patients without a chronic form of GvHD are characterized by the shorter survival time. This is also consistent with medical knowledge.

Another experiment concerned LAC dataset for which presented algorithm induced 3 survival rules. Each of them incorporate expression levels of 8 up to 10 genes. The analysis of Fig. 6 c confirms that obtained rules effectively distinguish patients’s survival rates on the basis of their expression profiles. The example survival rule has the following form:

- **R1**: *SLC20A1* <10.2∧*ITGA2* <8.7
    ∧*VEGF* <10.5∧*REG1A* <10.8
    ∧*SLC2A1* <8.9∧*SCGB2A2* <8.1
    ∧*S100P* ≥8.7∧*ATP2B1* <9.9

When applied on PTC dataset, LR-Rules generated 16 rules. Most common attributes were *BRAFV600E_RAS_score* and *nmut_CpGT* (11 and 9 occurrences, respectively) which had been previously associated with thyroid cancer development [49]. Selected survival rules are presented below.

- **R1**: *nmut_CpGT* ≥4.5
    ∧*mRNA_cluster_number* =5
    ∧*BRAFV600E_RAS_score* ∈(−0.976,−0.698)
- **R2**: *RPPA_cluster_number* =3
    ∧*mRNA_cluster_number* =5
    ∧*BRAFV600E_RAS_score* <−0.868
- **R4**: *meth_cluster* = classical 2
    ∧*Arm_SCNA_cluster* = Quiet
    ∧*miRNA_cluster_number* =6
    ∧*nmut_CpGT* <5.5
    ∧*BRAFV600E_RAS_score* ∈(−0.974,−0.889)
- **R8**: *Arm_SCNA_Cluster* = Quiet
    ∧*nmut_CpGT* ≥1.5
    ∧*BRAFV600E_RAS_score* ≥0.573
- **R14**: *nmut_CpGT* <6.5
    ∧*BRAFV600E_RAS_score* ∈[0.676,0.919)

As Fig. 6 d shows, the corresponding survival curves differ noticeably. Obtained rules model complex relationships between attributes and their influence on the survival time. For instance, BRAF^V600E^ and RAS were proven to be driver genes in many cancers including PTC [62]. Nevertheless, the effect of mutations in those genes on probability of recurrence is altered by other attributes. Particularly, BRAF-like tumors (those characterized by low values of *BRAFV600E_RAS_score*) may differ significantly in survival rate (compare R1, R2, and R4 rules). The same situation was in the case of RAS-driven cancers (rules R8 and R14).

## Conclusions

The experiments confirmed LR-Rules to perform significantly better than the KM estimator and similarly to survival trees CTREE and RPART in terms of prediction error. The comparison of LR-Rules and CW-Rules shows that the latter tends to get lower IBS values than our algorithm. This, however, is obtained at the cost of model complexity: CW-Rules always generated more rules than the competitors. In contrast, LR-Rules produces compact sets of rules of similar size as the tree models CTREE and RPART.

Superior performance and model comprehensibility make LR-Rules an effective alternative or a complement to survival trees, such as CTREE and RPART. Although every tree may be presented as a set of rules, the divide-and-conquer strategy used for tree construction usually leads to different rule sets than those generated by LR-Rules employing separate-and-conquer approach. In accordance with the strategy of tree building, every observation can be covered by exactly one rule, while the covering approach used by LR-Rules allows observations to be covered by multiple rules. The absence of this restrictive limitation in the presented algorithm may lead to the discovery of new or stronger patterns than those found by survival trees.

A characteristic feature of rule sets derived form a tree is the redundancy of conditions, particularly of the initial one that appears in every rule. In contrast, LR-Rules has the ability to induce rules with unique attributes. For example, when analysing BMT-Ch set, our algorithm generated a rule with only one condition: “*extcGvHD* = No”. In order to derive such a rule from a tree, an attribute *extcGvHD* would have to appear in the root, and thus, all the other rules would have to also take it into account.

An important advantage of the rule sets returned by the LR-Rules algorithm is also the fact that each rule can be considered independently from the others. This feature can be useful if modification of the generated rules is required, for example, in order to reflect the domain knowledge in a better way. The rules automatically generated by LR-Rules may constitute an initial set of hypotheses for the analyst. The expert, by adding/removing the rule conditions or by modifying their ranges, is allowed to carry out the different variants of the analysis. New rules can also be added to an existing set straightforwardly. In contrast, trees have to be treated as a whole in order to preserve the disjointed nature of the rules. Thus, the change of a condition in a node involves the need for modification of the conditions in all of its child nodes. Similarly, adding new rule to the tree requires its reconstruction.

We expect that the importance of survival analysis in medicine and biology will increase due to dissamination of high througput sequencing. Establishing how patients’ survival rate is affected by the presence of genetic variants, DNA methylation, or expressions of genes, microRNAs, and proteins will become of centeral interest. The application of LR-Rules on LAC and PTC datasets revealed interesting dependencies between genome/transcriptome/proteome-related features and their influence on the survival.

One of the greatest challenges to be faced when analyzing bioinformatics data is excessive dimensionality. High throughput technologies are able to produce hundreds of thousands of raw attributes which is prohibitive for machine learning strategies. Therefore, the application of all investigated survival analysis algorithms including LR-Rules has to be preceded by dimensionality reduction phase, i.e., construction, extraction and/or selection of features.

## Additional file

Additional file 1 Supplementary file. **Table S1**: IBS scores for different *mincov* values, **Table S2**: Number of rules for different *mincov* values, **Table S3**: IBS scores for different algorithms, **Table S4**: Number of rules for different algorithms, **Figure S1**: CD-diagrams comparing different *mincov* values with respect to IBS, **Figure S2**: CD-diagrams comparing different *mincov* values with respect to the number of rules, **Figure S3**: CD-diagrams comparing algorithms with respect to the number of rules. (PDF 875 kb)

## Abbreviations

BS: Brier score
CD diagram: Critical difference diagram
CNA: Copy number alteration
DnC: Divide-and-conquer
GvHD: Graft versus host disease
IBS: Integrated Brier score
KM: Kaplan-Meier
LAD: Logical analysis of data
PI: Prognostic index
RPPA: Reverse phase protein arrays
SnC: Separate-and-conquer
SNP: Single nucleotide polymorphism
SVM: Support vector machines
