# Supplementary material for: Learning rule sets from survival data
Source: BMC Bioinformatics. 2017 May 30;18:285. doi: 10.1186/s12859-017-1693-x (PMC5450332; doi:10.1186/s12859-017-1693-x)
Supplement: Additional file 1 — Supplementary file. Table S1: IBS scores for different mincov values, Table S2: Number of rules for different mincov values, Table S3: IBS scores for different algorithms, Table S4: Number of rules for different algorithms, Figure S1: CD-diagrams comparing different mincov values with respect to IBS, Figure S2: CD-diagrams comparing different mincov values with respect to the number of rules, Figure S3: CD-diagrams comparing algorithms with respect to the number of rules. (PDF 875 kb) [file 12859_2017_1693_MOESM1_ESM.pdf]

Learning rule sets from survival data
Supplementary results

Łukasz Wróbel[1], Adam Gudyś[1], Marek Sikora[2]

May 16, 2017

1. Institute of Informatics, Silesian University of Technology, Akademicka 16, 44-100 Gliwice, Poland

2. Institute of Innovative Technologies, EMAG, Leopolda 31, 40-189 Katowice, Poland

Corresponding Author: Łukasz Wróbel, lukasz.wrobel@polsl.pl

Table S1: The values of the integrated Brier score (IBS) evaluated on 18 datasets for different values of the *mincov* parameter of the LR-Rules algorithm.

	mincov						
dataset	(7)	(6)	(5)	(4)	(3)	(2)	(1)
actg320	0.0597	0.0599	0.0600	0.0602	0.0599	0.0612	0.0656
BMT-Ch	0.2231	0.2205	0.2239	0.2277	0.2185	0.2653	0.2485
cancer	0.1538	0.1539	0.1536	0.1525	0.1530	0.1543	0.1574
follic	0.1896	0.1873	0.1868	0.1883	0.1879	0.1884	0.1870
GBSG2	0.1783	0.1797	0.1776	0.1773	0.1773	0.1782	0.1787
hd	0.2185	0.2181	0.2190	0.2185	0.2185	0.2190	0.2193
LAC	0.2116	0.2203	0.2056	0.2096	0.2192	0.2197	0.2270
lung	0.1472	0.1476	0.1476	0.1479	0.1480	0.1480	0.1486
Melanoma	0.1823	0.1798	0.1792	0.1746	0.1768	0.1731	0.1733
mgus	0.1719	0.1721	0.1723	0.1712	0.1706	0.1703	0.1662
PTC	0.1426	0.1462	0.1435	0.1415	0.1463	0.1448	0.1321
pbc	0.1502	0.1510	0.1522	0.1532	0.1542	0.1524	0.1552
std	0.2220	0.2224	0.2212	0.2208	0.2218	0.2205	0.2220
uis	0.1539	0.1530	0.1539	0.1531	0.1531	0.1532	0.1577
wcgs	0.0432	0.0429	0.0430	0.0429	0.0430	0.0435	0.0453
whas1	0.2130	0.2125	0.2126	0.2119	0.2130	0.2141	0.2137
whas500	0.2046	0.2042	0.2038	0.2049	0.2044	0.2045	0.2041
zinc	0.0931	0.0926	0.0935	0.0937	0.0937	0.0938	0.0938

Table S2: The number of rules obtained for 18 datasets for different values of the *mincov* parameter of the LR-Rules algorithm.

	mincov						
dataset	(7)	(6)	(5)	(4)	(3)	(2)	(1)
actg320	13.1	13.7	14.6	15.4	18.8	25.5	70.1
BMT-Ch	4.3	4.5	5.1	6.4	9.7	15.6	19.2
cancer	7.3	7.7	8.3	8.8	10.4	13.7	23.5
follic	5.0	5.4	5.8	5.8	6.5	6.4	7.3
GBSG2	7.7	7.7	8.9	9.7	10.0	11.3	12.0
hd	4.6	4.7	4.6	4.9	4.9	5.2	5.9
LAC	2.8	3.2	3.6	4.1	5.1	6.7	10.1
lung	5.1	5.2	5.3	5.4	5.5	5.7	6.8
Melanoma	6.2	6.8	7.2	7.6	8.3	9.1	11.1
mgus	7.4	7.6	7.9	8.2	9.3	9.4	10.8
PTC	18.7	21.2	23.2	25.0	27.6	31.5	22.8
pbc	3.7	3.7	3.9	4.0	4.3	5.0	6.4
std	17.8	17.6	18.8	20.5	22.4	25.2	32.8
uis	5.5	5.9	6.2	7.1	8.2	11.2	39.6
wcgs	21.8	22.9	25.9	30.4	39.0	64.9	178.7
whas1	3.8	3.8	4.0	3.9	4.1	4.4	5.2
whas500	4.6	4.8	5.0	5.3	5.5	6.4	6.6
zinc	7.1	7.8	8.8	9.7	10.7	13.5	13.1

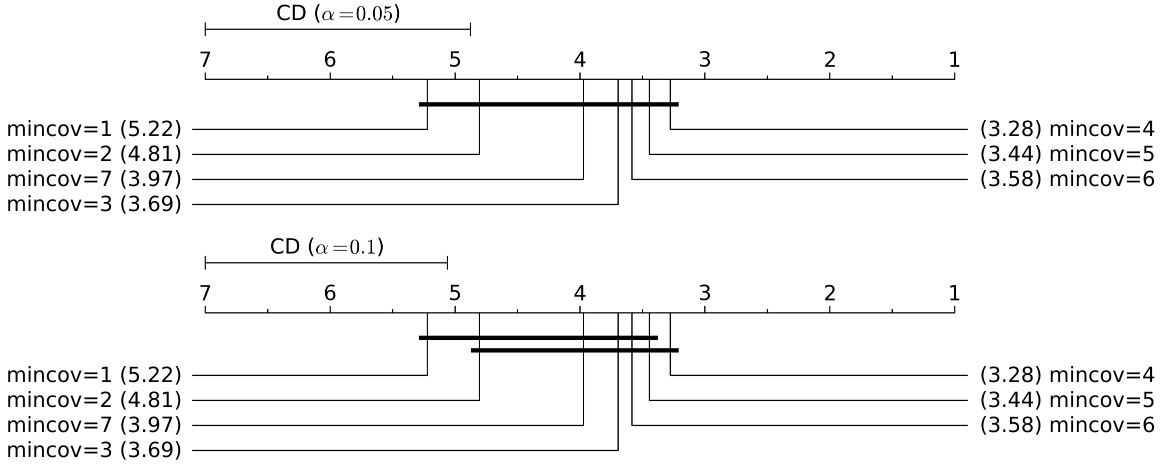

Figure S1: CD diagrams comparing the results of the LR-Rules algorithm with different values of the *mincov* parameter (1 to 7) in terms of the integrated Brier score (IBS) on the level of significance of 0.05 (upper diagram) and 0.1 (lower diagram). The exact values of average ranks are given beside each parameter in the parentheses. A group of parameters, which are not statistically different at the given level of significance, are connected with bold line.

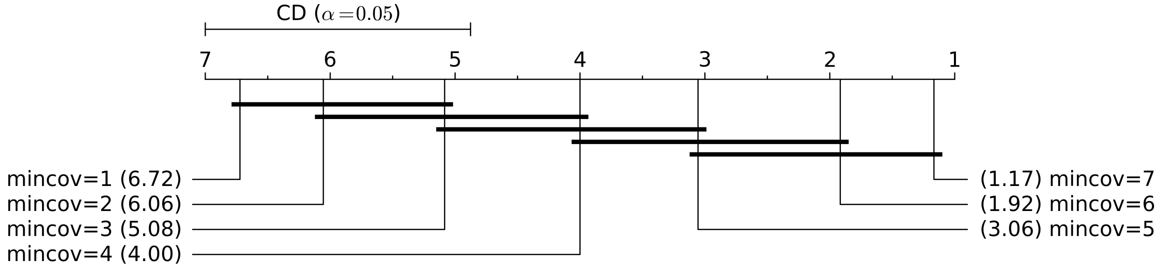

Figure S2: CD diagram comparing the results of the LR-Rules algorithm with different values of the *mincov* parameter (1 to 7) in terms of the number of the generated rules. The exact values of average ranks are given beside each parameter in the parentheses. Groups of parameters which are not statistically different at 0.05 significance level are connected with bold line.

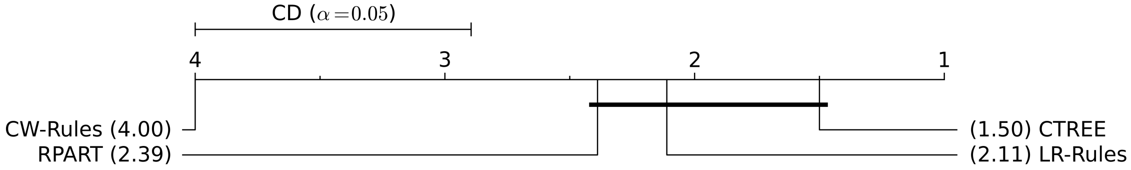

Figure S3: CD diagram comparing the LR-Rules, CW-Rules, CTREE and RPART algorithms in terms of the size of the rule-models. In brackets next to each algorithm, an average value of rank is given. A group of algorithms which are not statistically different at 0.05 significance level are connected with bold line.

Table S3: Comparison of LR-Rules, CW-Rules, CTREE, RPART, and the KM estimator with respect to the integrated Brier score (IBS) on 18 datasets.

dataset	LR-Rules	CW-Rules	CTREE	RPART	KM
actg320	0.0597	0.0581	0.0595	0.0599	0.0614
BMT-Ch	0.2231	0.2148	0.2062	0.2410	0.2386
cancer	0.1538	0.1540	0.1553	0.1709	0.1538
follic	0.1896	0.1838	0.1891	0.1863	0.2022
GBSG2	0.1783	0.1666	0.1796	0.1778	0.1935
hd	0.2185	0.2171	0.2085	0.2102	0.2326
LAC	0.2116	0.1884	0.1946	0.2284	0.2063
lung	0.1472	0.1444	0.1430	0.1431	0.1686
Melanoma	0.1823	0.1651	0.1818	0.1820	0.1911
mgus	0.1719	0.1709	0.1701	0.1833	0.1903
PTC	0.1426	0.1340	0.1485	0.1604	0.1285
pbc	0.1502	0.1450	0.1547	0.1713	0.1965
std	0.2220	0.2187	0.2219	0.2239	0.2208
uis	0.1539	0.1576	0.1537	0.1541	0.1713
wcgs	0.0432	0.0423	0.0436	0.0439	0.0443
whas1	0.2130	0.2015	0.1942	0.1969	0.2368
whas500	0.2046	0.1840	0.1890	0.1909	0.2373
zinc	0.0931	0.0955	0.1044	0.1110	0.1097

Table S4: Comparison of LR-Rules, CW-Rules, CTREE and RPART algorithms with respect to the number of rules on 18 datasets.

dataset	LR-Rules	CW-Rules	CTREE	RPART
actg320	13.1	190.5	4.0	6.7
BMT-Ch	4.3	20.5	4.5	11.4
cancer	7.3	29.0	3.0	9.5
follic	5.0	75.2	4.3	5.4
GBSG2	7.7	120.5	4.0	6.3
hd	4.6	117.6	2.9	2.4
LAC	2.8	16.6	2.2	5.3
lung	5.1	54.3	5.2	4.2
Melanoma	6.2	30.8	2.8	9.9
mgus	7.4	27.1	5.1	10.2
pbc	3.7	75.3	6.4	13.6
PTC	18.7	80.8	3.5	5.4
std	17.8	160.6	3.6	2.8
uis	5.5	68.7	5.1	11.6
wcgs	21.8	664.2	9.6	5.7
whas1	3.8	63.6	6.3	6.8
whas500	4.6	82.8	5.8	11.3
zinc	7.1	46.2	2.3	9.5
